# Impairment of the immune response after transcuticular introduction of the insect gonadoinhibitory and hemocytotoxic peptide *Neb*-colloostatin: A nanotech approach for pest control

**DOI:** 10.1038/s41598-019-46720-9

**Published:** 2019-07-17

**Authors:** Elżbieta Czarniewska, Patryk Nowicki, Mariola Kuczer, Grzegorz Schroeder

**Affiliations:** 1 Department of Animal Physiology and Development, Institute of Experimental Biology, Faculty of Biology, Adam Mickiewicz University in Poznań, Uniwersytetu Poznańskiego str. 6, 61-614 Poznań, Poland; 2 Faculty of Chemistry, University in Wrocław, F. Joliot-Curie str. 14, 50-383 Wrocław, Poland; 3 Faculty of Chemistry, Adam Mickiewicz University in Poznań, Uniwersytetu Poznańskiego str. 8, 61-614 Poznań, Poland

**Keywords:** Peptides, Innate immunity

## Abstract

This article shows that nanodiamonds can transmigrate through the insect cuticle easily, and the doses used were not hemocytotoxic and did not cause inhibition of cellular and humoral immune responses in larvae, pupae and adults of *Tenebrio molitor*. The examination of the nanodiamond biodistribution in insect cells demonstrated the presence of nanodiamond aggregates mainly in hemocytes, where nanoparticles were efficiently collected as a result of phagocytosis. To a lesser extent, nanodiamond aggregates were also detected in fat body cells, while they were not observed in Malpighian tubule cells. We functionalized nanodiamonds with *Neb*-colloostatin, an insect hemocytotoxic and gonadoinhibitory peptide, and we showed that this conjugate passed through the insect cuticle into the hemolymph, where the peptide complexed with the nanodiamonds induced apoptosis of hemocytes, significantly decreased the number of hemocytes circulating in the hemolymph and inhibited cellular and humoral immune responses in all developmental stages of insects. The results indicate that it is possible to introduce a peptide that interferes with the immunity and reproduction of insects to the interior of the insect body by means of a nanocarrier. In the future, the results of these studies may contribute to the development of new pest control agents.

## Introduction

Due to the excellent physical and chemical properties of nanodiamonds (NDs), such as their small size, hardness, large surface area, high absorption capacity and chemical stability, various ND applications in the field of nanobiotechnology have been proposed (e.g., drug delivery, use as biosensors and in bioimaging and coating of implants)^1–4^. NDs are considered biocompatible; however, the biomedical use of these nanoparticles is now being discussed because some recent works have shown the adverse effects of NDs on insect, chicken embryo, mouse and rat cells^2,5–9^. However, few studies have focused on ND cytotoxicity in animal models *in vivo*^2,5,10–15^.

The insect immune system can be a target for the development of novel strategies for suppressing pest vitality. A pleiotropic insect peptide hormone that disrupts oogenesis and exerts a hemocytotoxic effect in insects may be a candidate for use as a bioinsecticide. One such peptide is *Neb*-colloostatin (SIVPLGLPVPIGPIVVGPR), an oostatic peptide isolated from the ovaries of the gray flesh fly *Neobellieria bullata*^16^. Upon injection, this peptide inhibits ovarian development in the *Tenebrio molitor* beetle, delaying oviposition and reducing the number of eggs laid by the females^17^. Nanomolar doses of *Neb*-colloostatin induce atresia in the *T. molitor* ovary. The hormone causes degeneration of mealworm ovarian follicles, including changes in the morphology and viability of follicular cells, and oosorption occurs as a consequence of these changes^18^. *Neb*-colloostatin also induces apoptosis in the hemocytes of *T. molitor*, as visualized by changes in morphology and viability of these cells^19^. Moreover, our structure-activity analysis of new *Neb*-colloostatin analogs in *T. molitor* hemocytes revealed pro-apoptotic analogs that were more potent than the native peptide^20^. However, *Neb*-colloostatin and its more active analogs cannot be introduced into the insect body *per os* because, similar to other peptides and proteins, they would be immediately degraded by peptidases in the insect midgut. Therefore, it is a challenge to introduce a peptide molecule into the hemocoel through the cuticle to induce cytotoxic effects in insects. Several studies have provided evidence of successful penetration of the cuticle by amphiphilic analogs of insect pyrokinin neuropeptides^21–25^, and these studies have inspired us to use NDs for this purpose.

The insect cuticle is a natural biocomposite with unique physiochemical properties that protects insects from outside influences and pathogen infection. It is composed of an apolar waxy layer over an apolar protein and chitin matrix^26^. However, there are fine lipid-coated pore canals running through the insect cuticle at right angles to the surface, ranging from 6–65 nm in diameter in the *T. molitor* beetle, which allow insects to detect and secrete pheromones or other physiological substances^27,28^. Thus, the porosity of the insect cuticle may be exploited to introduce biologically active molecules causing pathophysiological effects in the insect to induce insecticidal effects.

The mealworm beetle, *T. molitor*, is a model insect that is frequently used for prospective screening of the insecticidal activities of chemicals^17–20,29–33^. In this work, we examined the *in vivo* effect of NDs on the cellular and humoral immune responses (viability and number of circulating hemocytes, phagocytosis, nodule formation and phenoloxidase activity) of larvae, pupae and imagos of *Tenebrio molitor* to determine whether these nanoparticles are cytotoxic to immunocompetent cells. We also investigated the distribution of NDs in insect tissues. The results from this part of the study were crucial for further research aimed at introducing the active insect peptide *Neb*-colloostatin into the insect body *via* a transcuticular route to induce immunoinhibitory effects in *Tenebrio molitor*. We demonstrated that NDs—including NDs coupled with *Neb*-colloostatin—can penetrate the insect cuticle. In various *in vivo* bioassays, we showed that NDs introduced into the hemocoel of the insect through the cuticle did not change the viability of hemocytes and did not interfere with the innate immune response of the *T. molitor* larvae, pupae or adults. Furthermore, *Neb*-colloostatin introduced into the hemocoel in complexes with NDs retained its specific hemocytotoxic activity and disturbed the cellular and humoral immune responses of all developmental stages of the *T. molitor* beetle.

## Materials and Methods

### Functionalization of NDs

Chemicals: NDs in nanopowder form with a size <10 nm were acquired from Sigma-Aldrich (636444 Sigma-Aldrich). Fluorescein isothiocyanate (FITC) from Sigma-Aldrich **(**F7250 Sigma-Aldrich**)** was used to prepare the fluorescent NDs as detailed below. *Neb*-colloostatin was synthesized by the classical solid-phase method^34,35^. Other reagents were purchased from IRIS Biotech, Fluka, and Sigma-Aldrich.

### Fluorescent labeling of NDs with FITC

The first step in the functionalization of the NDs was acid oxidation of their surfaces, followed by reaction with FITC. In this procedure, 0.5 g of ND powder was treated with 10 mL of a 3:1 (v/v) H_2_SO_4_:HClO_4_ mixture at 40 °C for 2 hours. Then, the NDs were isolated by centrifugation. Next, the NDs were washed with deionized water and centrifuged. The NDs were dried for 24 hours at 40 °C. The thus-prepared NDs were reacted with FITC in acetone; 0.02 g of FITC in 10 mL of acetone was added to a suspension of 0.1 g of NDs in 10 mL of acetone. The mixture was stirred at 40 °C for 12 hours. Functionalized NDs (FITC-ND) were centrifuged, washed with acetone, and dried at 40 °C. The NDs showed fluorescence characteristic of fluorescein.

### Bioconjugation of ND and *Neb*-colloostatin

The noncovalent interaction between the ND surface and the peptide was used for the physical functionalization of NDs for *Neb*-colloostatin. A preparation of 0.01 g of *Neb*-colloostatin was dissolved in a water/ethanol solution (1:1), and 0.05 g of NDs was added. The suspension was stirred for 2 hours. The ND-*Neb*-colloostatin system was centrifuged, washed, and vacuum-dried at 25 °C. Fourier transform infrared (FTIR) spectroscopy and thermogravimetric analysis (TGA) were used to test the material. The infrared spectra were obtained on an IFS 66 v/s Vacuum FT-IR spectrophotometer from Bruker equipped with an MCT detector (125 scans, 2 cm^−1^ resolution). The spectra were recorded in the 400–4000 cm^−1^ range for KBr pellets. To confirm the functionalization of NDs for the studied materials, TGA measurements were performed with a Setsys 1200 thermogravimetric analyzer (Setaram) at a heating rate of 10 °C min^−1^ from room temperature to 1000 °C under a helium atmosphere. Elemental analysis of NDs and ND-*Neb*-colloostatin was performed to determine the chemical composition of the functionalized NDs.

### Preparation of the ND solution

NDs, FITC-labeled NDs, and ND-*Neb*-colloostatin were dissolved in physiological saline for *Tenebrio* to yield stock solutions of 1 mg or 10 mg/mL. The prepared stock solutions were stored at −30 °C, and the working solutions were prepared in physiological saline just before use. The pure and functionalized ND solutions with different concentrations were sonicated for 2 hours before use to break up possible aggregates. Treatments were performed with NDs, FITC-NDs, and ND-*Neb*-colloostatin solutions at different concentrations.

### Insects

A stock culture of *T. molitor* was maintained at the Department of Animal Physiology and Developmental Biology, Institute of Experimental Biology, Adam Mickiewicz University, Poznań, Poland. All beetles used in our experiments were derived from parents that were less than 1 month old. The control and experimental groups (larvae, pupae and adults) were maintained at the same population density in separate plastic boxes in a climate chamber at a constant temperature of 26 °C, with a relative humidity of 60 ± 5% and a photoperiod of 12 hours of light and 12 hours of darkness. Studies were carried out on fifteen or thirty insects for each treatment.

### Hemocytic bioassays with NDs and ND-*Neb*-colloostatin

#### Examination of ND passage through the insect cuticle

In this experiment, we used fluorescence-labeled NDs to investigate whether these nanoparticles would be phagocytosed by hemocytes after they were injected into the hemolymph or when applied to the surface of the cuticle. Four-day-old *T. molitor* were split into two control groups and two treatment groups and were anesthetized, disinfected with 70% ethanol and washed in distilled water before saline or FITC-NDs treatment. The first control group of beetles was injected with 2 µL of physiological saline, whereas the first experimental group was injected with 2 µL of FITC-ND solution, administering a dose of 2 ng of FITC-NDs per insect. The second control group of beetles topically received 5 µL of physiological saline on the surface of the cuticle, whereas the second experimental group topically received 5 µL of FITC-ND solution at a dose of 5 µg of NDs per insect. Hemolymph was collected from the insects 2 hours after injection or 1 day after topical treatment. The injection, hemolymph collection and hemocyte preparation procedures were carried out according to previously described methods^19^. To visualize the cell nuclei of hemocytes, the cells were stained with propidium iodide (Sigma-Aldrich P4170) solution for 5 min in the dark. The preparations of hemocytes were examined under a Zeiss LSM 510 confocal laser scanning microscope (in fluorescence and transmitted light) to detect the presence of FITC-NDs in the interior of hemocytes. Each experimental group consisted of fifteen insects.

#### Hemocytotoxicity assay

To evaluate whether NDs and ND-*Neb*-colloostatin induce apoptosis in *T. molitor* hemocytes, anaesthetized 4-day-old insects received 5 µL of ND solution topically on the cuticle containing a dose of 5 µg or 41.5 µg of NDs per insect, or they received ND-*Neb*-colloostatin solution with a dose of approximately 4.15 µg of NDs and 0.85 µg of *Neb*-colloostatin or 41.5 µg of NDs and 8.5 µg of *Neb*-colloostatin per insect. Control insects topically received physiological saline or *Neb*-colloostatin solution with a dose of 0.85 or 8.5 µg of peptide per insect. Samples of hemolymph from control and test insects were collected 1 day after the application of the tested compounds. Hemocytes were prepared and then used to detect active caspases, F-actin microfilaments and DNA according to previously described methods^19^. For this purpose, hemocytes were stained with an inhibitor of caspase activity (a sulforhodamine derivative of valylalanyl aspartic acid fluoromethyl ketone, SR-VAD-FMK; sulforhodamine multicaspase (1–9) activity kit AK-115, BIOMOL, Plymouth Meeting, PA, USA) along with Oregon Green 488–phalloidin (ThermoFisher Scientific) to visualize the F-actin cytoskeleton and DAPI to visualize cell nuclei. In brief, adherent hemocytes were incubated in reaction medium (1/3× SR-VAD-FMK) for 30 min at room temperature in the dark, rinsed three times with wash buffer for 5 min each at room temperature and finally fixed in 3.7% paraformaldehyde for 10 min and permeabilized in 0.1% Triton X-100 for 5 min at room temperature. Hemocytes were washed in physiological saline and stained with Oregon Green 488–phalloidin for 20 min at room temperature in the dark. After washing again in physiological saline, the hemocytes were stained with DAPI solution for 5 min at room temperature in the dark. The induction of apoptosis in hemocytes was examined under a Nikon Eclipse TE 2000-U fluorescence microscope. Each experimental group consisted of fifteen insects.

#### Immune response assays

After anesthesia, *T. molitor* beetles at various stages of development (larvae, pupae and 4-day-old adults) were split into control and experimental groups to investigate whether NDs and ND-*Neb*-colloostatin changed the cellular immune response (phagocytosis and nodulation), humoral immune response (phenoloxidase (PO) activity) and circulating hemocyte count (CHC). The control insects were injected with 2 µL of physiological saline, whereas the experimental insects were injected with 2 µL of ND solution containing a dose of 2 ng of NDs per insect or 2 µL of ND-*Neb*-colloostatin solution containing a dose of approximately 1.66 ng of NDs and 0.34 ng of *Neb*-colloostatin, or they were topically treated with 5 µL of ND solution containing a dose of 5 µg of NDs per insect or 5 µL of ND-*Neb*-colloostatin solution containing a dose of approximately 4.15 µg of NDs and 0.85 µg of *Neb*-colloostatin. Each control and experimental group consisted of fifteen insects for each assay.

#### Testing the circulating hemocyte count

After anesthesia, the control and ND- and ND-*Neb*-colloostatin-treated larvae, pupae and adults were washed in 70% ethanol and rinsed in distilled water. To determine the CHC, the collected hemolymph sample (2 µL) was diluted in 18 µL of physiological saline (274 mmol/L NaCl, 19 mmol/L KCl, 9 mmol/L CaCl_2_) containing an anticoagulant buffer (4.5 mmol/L citric acid and 9 mmol/L sodium citrate, 5:1 v/v). Subsequently, the hemolymph samples were placed on a Bürker hemocytometer and analyzed under a light microscope. The CHC value was based on the number of hemocytes observed in 15 random squares and the diluted hemolymph sample. The CHC value was analyzed for each group of beetles.

#### Phagocytosis

The phagocytic ability of hemocytes isolated from the control and ND- and ND-*Neb*-colloostatin-treated insects at various stages of development was studied *in vivo* with latex beads (Sigma-Aldrich L1030). Latex beads suspended in physiological saline (1:500 v/v) were injected into beetles 2 hours after ND or ND-*Neb*-colloostatin injection or 1 day after ND or ND-*Neb*-colloostatin topical application. Hemolymph samples were collected 1 hour after the injection of a latex bead solution and incubated for 30 min on coverslips coated with poly-L-lysine at room temperature in the dark. Subsequently, the hemocytes were washed with saline, fixed in 3.7% paraformaldehyde for 10 min and stained with DAPI solution for 5 min in the dark to visualize nuclei. The prepared hemocytes were used for phagocytosis analysis under a Nikon Eclipse TE 2000 U fluorescence microscope. Photos of hemocytes were taken with a Nikon DS-1QM digital camera and analyzed with NIS Elements AR 3.10 software. Data are expressed as the percentage of phagocytic cells among the total number of hemocytes in the image.

#### Nodulation

Nodule formation was studied according to a previously described method^32^. In brief, two hours after the injection or topical application of ND and ND-*Neb*-colloostatin solutions, beetles at various stages of development were anaesthetized again, washed in distilled water and disinfected and then injected with *Staphylococcus aureus* solution in physiological saline (1:500 v/v; 5 μL, formalin-fixed suspension of essentially nonviable *S. aureus*; Sigma R S2014 Saint Louis, Missouri, USA). Positive control insects were injected with only *S. aureus* solution in physiological saline (5 μL). Nodule formation was measured on the third day of the experiment. Beetles were dissected to expose the nodules on the dorsal side of the hemocoel. The insect body was pinned to a SYLGARD R silicone-filled Petri dish, and the hemocoel was washed with physiological saline. Next, the fat body and alimentary canal were removed. The number of nodules was studied with an Olympus SZX 12 stereoscopic microscope (Olympus Co., Tokyo, Japan), and 3 images of each beetle were taken with an Olympus U-LH100HG digital camera (Olympus Co., Tokyo, Japan). The number of nodules on the dorsal side of the hemocoel was counted in all of the beetles.

#### Humoral immune response

PO activity in *S. aureus*-infected larvae, pupae and adults of *T. molitor* was studied according to a modified method described previously^36,37^. In brief, 1 μL of hemolymph was placed on white filter paper (Whatman No. 52) soaked in 10 mM sodium phosphate buffer containing 2 mg/mL DL-DOPA (Sigma Aldrich). Subsequently, the filter papers with the samples were incubated for 30 min at room temperature and air-dried. The samples were then scanned with a SHARP AR 153E N digital laser copier/printer (300 dpi, 8 bits, grayscale), and the images were analyzed with ImageJ software (ver. 2). PO activity assessment was based on the mean pixel value of the images.

#### Biodistribution of NDs

To evaluate the tissue distribution of NDs, we applied FITC-NDs topically to the cuticles (at a dose of 5 µg of FITC-NDs per insect) of 4-day-old beetles. One day after the administration of FITC-NDs on the cuticle, we isolated the fat body and Malpighian tubules in cold physiological saline under a stereomicroscope. Immediately after the isolation of tissues, the physiological fluid was gently drawn off with tissue paper. The fresh, unfixed fat body and Malpighian tubules were placed in a drop of OCT Embedding Matrix (Cellpath^™^) and were quickly frozen at −20 °C, after which 10 µm-thick tissue sections were cut with a cryostat (Leica CM 1850) at −20 °C and immediately transferred to a room-temperature microscope slide coated with poly-L-lysine. Subsequently, the tissue sections were fixed in 3.7% paraformaldehyde for 10 min at room temperature. The preparations of fat body and Malpighian tubules were washed in physiological solution, mounted using mounting medium, and examined under a Zeiss LSM 510 confocal laser scanning microscope to detect the presence of FITC-NDs in cells.

#### Statistical analysis

For statistical analysis of data, we used Graph Pad Prism 5 software. Statistical analyses were performed using Student’s *t*-test. All data were considered statistically significant at a p-value < 0.05.

## Results

### Chemical analysis of ND functionalization

**Figure 1:**
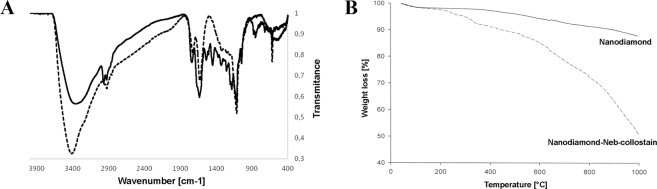
Chemical analysis of ND-*Neb*-colloostatin. (**A**) FTIR spectra of ND (−) and ND-*Neb*-colloostatin (- - -) and (**B**) TG curves of ND and ND-*Neb*-colloostatin.

As shown in Fig. 1A, the FTIR spectrum of the NDs presented characteristic bands at 3413, 2931, 2083, 1729, 1603, 1116, and 618 cm^−1^. In contrast, the spectrum of ND-*Neb*-colloostatin exhibited bands at 3359, 2924, 2965, 1739, 1642, 1539, 1447, 1332, 1254, 1181, 854, and 620 cm^−1^. For the functionalized material spectrum, bands characteristic of the peptide were observed in the 1300–1700 cm^−1^ range.

Figure 1B shows the TG curve of ND-*Neb*-colloostatin in comparison with that of the NDs, revealing increased weight loss of 40.2% at 1000 °C; this result was correlated with the thermal decomposition of the NDs. The increase in weight loss in the case of the ND-*Neb*-colloostatin group was continuous with an increasing temperature and was associated with decomposition of *Neb*-colloostatin deposited on ND surfaces.

The elemental analysis revealed the chemical composition of the functionalized NDs. The results regarding the contents of C, N, and H in ND-*Neb*-colloostatin are presented in Table 1. In comparison with NDs alone, ND-*Neb*-colloostatin showed a decrease in the elemental content of C and an increase in the elemental contents of N and H.

**Table 1:** Elemental Analysis Data of NDs and ND-*Neb*-colloostatin.

Sample	Elemental composition (%)		
	C	N	H
NDs	87.75	2.31	1.15
ND-*Neb*-colloostatin	81.34	4.18	1.85

### Examination of ND penetration into the hemocoel of insects by the transcuticular route

**Figure 2:**
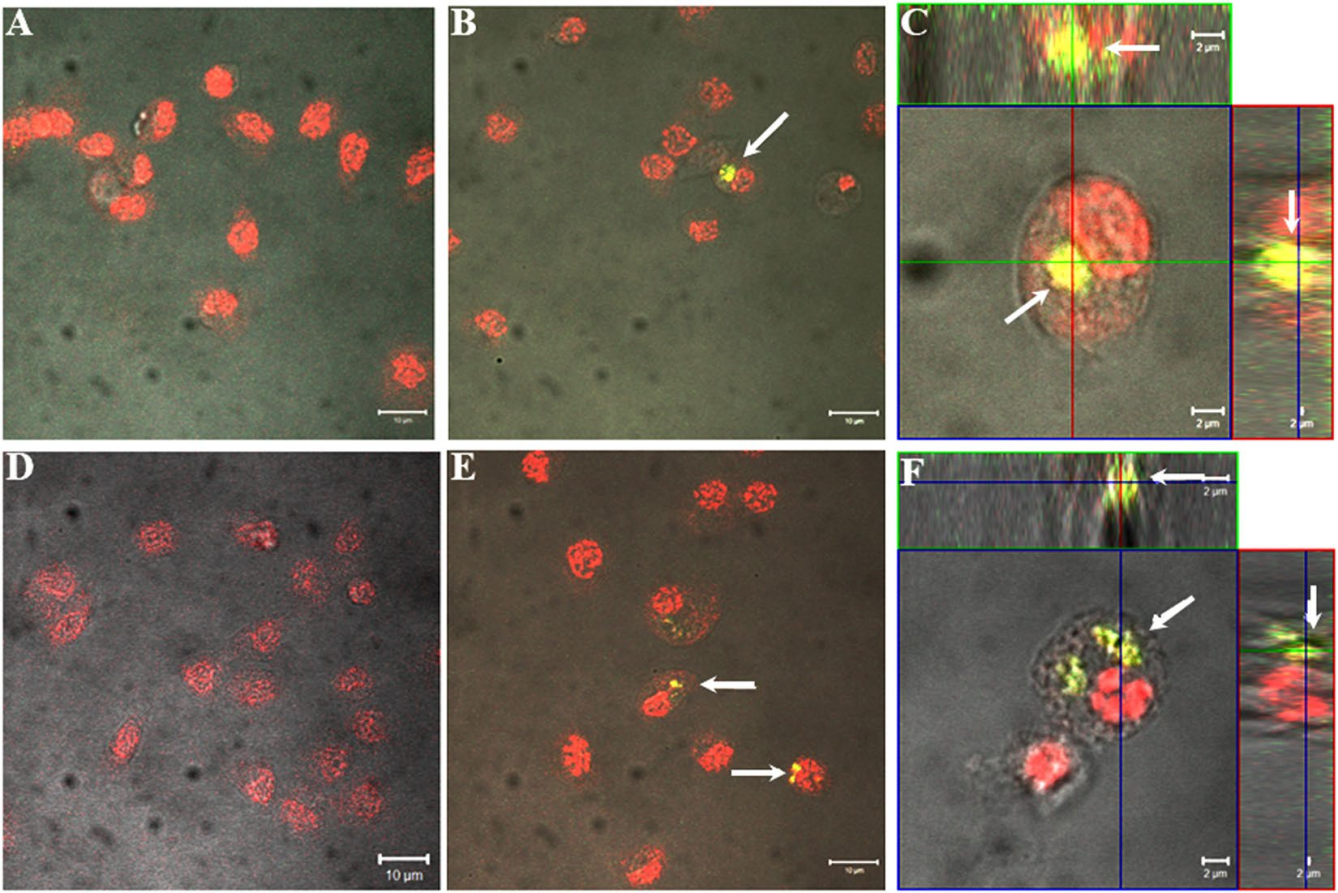
*In vivo* hemocytic test in *Tenebrio molitor* showing ND passage through the cuticle. Insect hemocytes after injection (**B,C**) and topical application (**E,F**) of FITC-NDs (at a dose of 2 ng or 5 µg of FITC-NDs per insect, respectively). Control beetles received physiological solution through injection (**A**) or topically (**D**). Regardless of the route of FITC-NDs administration to the beetles, they were visible inside the cytoplasm of hemocytes as yellow-green aggregates (arrows). Hemocyte nuclei were stained with propidium iodide solution. Scale bars: 10 µm (**A,B,D,E**) and 2 µm (**C,F**).

To investigate the penetration of NDs through the insect cuticle, we used a special feature of hemocytes: their ability to phagocytose abiotic targets. In this study, we used fluorescently labeled NDs, which were injected or applied topically on the insect cuticle. First, we showed that FITC-NDs were visible in hemocytes 2 hours after nanoparticle injection into the *T. molitor* hemocoel (Fig. 2B,C). This result indicated that FITC-NDs were phagocytosed by hemocytes and that aggregates of these particles could be detected inside these cells under a fluorescence confocal microscope. Second, 1 day after the topical application of FITC-NDs, we revealed FITC-ND aggregates in hemocytes (Fig. 2E,F).

### Hemocytotoxicity assay

**Figure 3:**
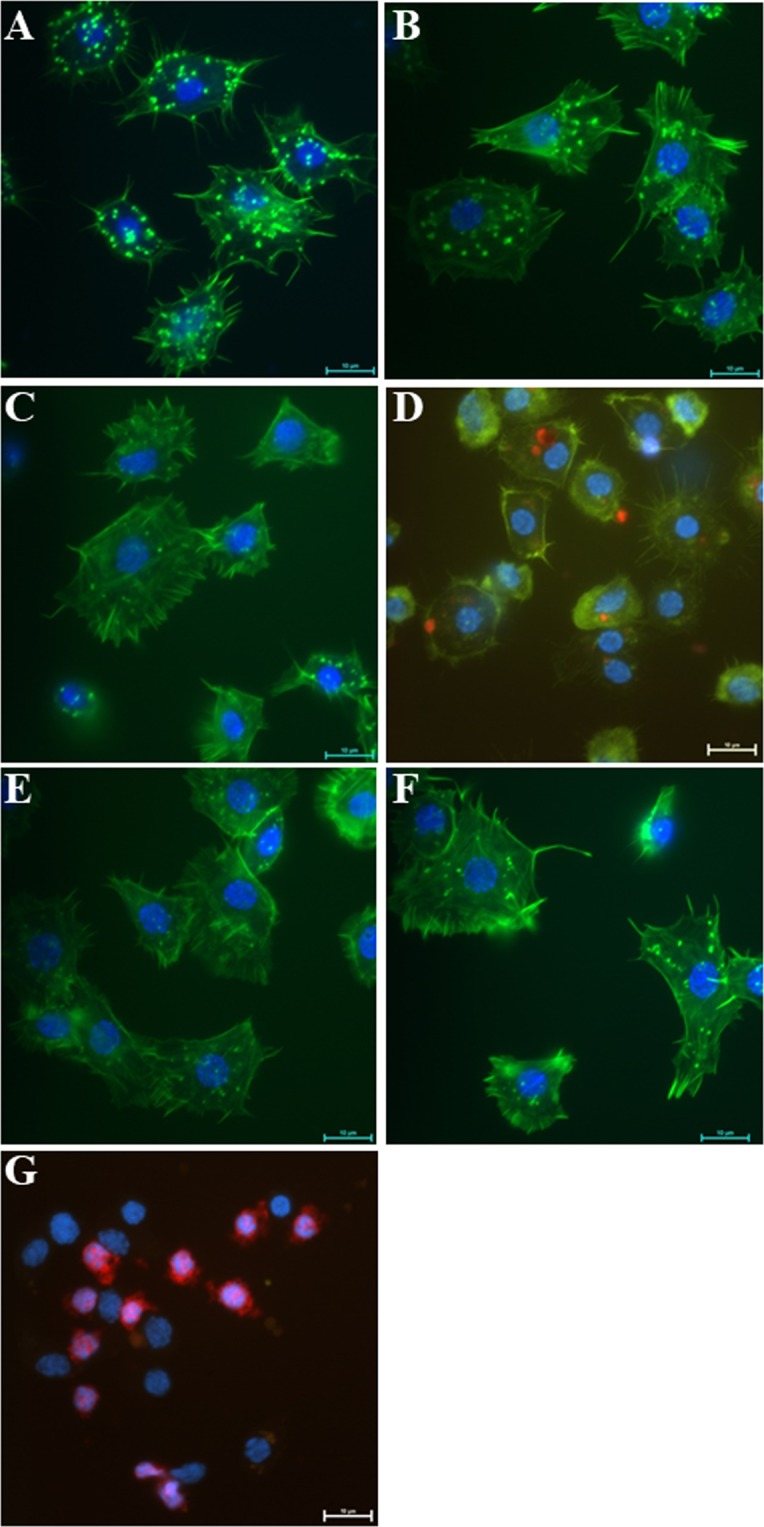
Effect of NDs and ND-*Neb*-colloostatin on the morphology and viability of *T. molitor* hemocytes. The hemocytes 1 day after the topical application of: NDs at a dose of (**B**) 5 µg or (**E**) 41.5 µg or ND-*Neb*-colloostatin at a dose of (**D**) approximately 4.15 µg of NDs and 0.85 µg of *Neb*-colloostatin or (**G**) 41.5 µg of NDs and 8.5 µg of *Neb*-colloostatin per insect. Control beetles topically received (**A**) physiological solution or *Neb*-colloostatin at a dose of (**C**) 0.85 µg or (**F**) 8.5 µg of peptide per insect. All hemocytes were stained with SR-VAD-FMK reagent for caspase activity detection (red color), with Oregon Green phalloidin for F-actin cytoskeleton staining (green color) and with DAPI for DNA staining (blue color).

The administration of NDs or *Neb*-colloostatin on the surface of the cuticle did not affect the hemocytes regardless of the applied dose of NDs or peptide (Fig. 3B,C,E,F). Hemocytes isolated from topically ND- or peptide-treated insects were viable, presented a normal morphology, exhibited high adhesion to coverslips, and formed a long filopodia. In these adherent cells, no changes in chromatin were visible, the cortical F-actin cytoskeleton was well formed, and no active caspases were observed. However, when ND-*Neb*-colloostatin was topically applied to the insects, it caused changes in the morphology and viability of hemocytes (Fig. 3D,G). In these hemocytes, we observed apoptosis; the apoptotic cells showed reduced adhesion to coverslips, formed shorter filopodia or exhibited no filopodia, and displayed alterations of the nuclei and F-actin cytoskeleton and caspase activation. The degree of changes in nuclei, F-actin depolymerization and caspase activation in hemocytes depended on the ND-*Neb*-colloostatin dose administered topically to the insects.

### CHC assay

**Figure 4:**
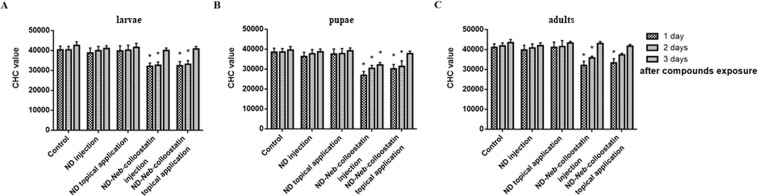
The circulating hemocyte count in *T. molitor* larvae (**A**), pupae (**B**) and adults (**C**) following the injection or topical application of NDs or ND-*Neb*-colloostatin.

Regardless of the method of ND application in insects, the number of hemocytes in the hemolymph of larvae, pupae and adults of *T. molitor* was very similar to the CHC value of the control groups throughout the experiment (Fig. 4). In the case of larvae from both ND-*Neb*-colloostatin-treated groups, we observed a significant decrease in the CHC value on the first and second days of study (Fig. 4A). In the case of pupae from the ND-*Neb*-colloostatin-injected group, we observed a significant reduction in the CHC value over the course of the entire experiment, whereas in the ND-*Neb*-colloostatin topically treated pupae, a significant decrease in the CHC value was observed on the first and second days of the study (Fig. 4B). In adults from the ND-*Neb*-colloostatin-injected group, significant changes were observed on the first and second days of the experiment, whereas in adults from the ND-*Neb*-colloostatin topically treated group, a significant reduction in the CHC value was observed only on the first day of the study (Fig. 4C).

### Cellular immune response

**Figure 5:**
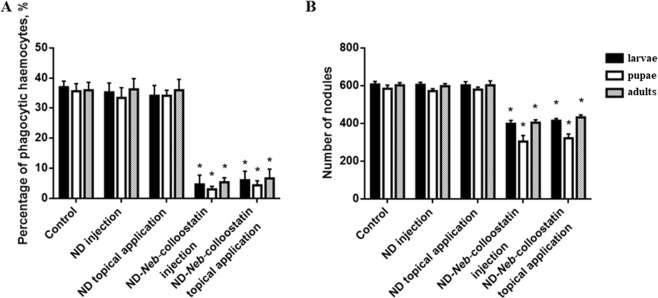
Phagocytosis (**A**) and nodule formation (**B**) in the *T. molitor* larvae, pupae and adults following the injection or topical application of NDs or ND-*Neb*-colloostatin. The values shown are the means ± S.D.s. Results that significantly differed from the control group at *p* < 0.05 are indicated (*).

The *in vivo* analysis of ND passage through the insect cuticle showed that FITC-NDs were phagocytosed by hemocytes and were aggregated inside these cells (Fig. 2). In the experiment showing the influence of NDs on the phagocytosis of another abiotic target, we showed that at a dose of 2 ng or 5 µg of NDs per insect (injected or applied to the surface of the cuticle, respectively), these nanoparticles did not impair the phagocytosis of latex beads (Fig. 5A). Irrespective of the application method of NDs, no significant changes in the phagocytic activity of hemocytes were observed in larvae, pupae and adults, and the percentages of phagocytic hemocytes were very similar to those in the control groups.

However, the injection of ND-*Neb*-colloostatin solution (at a dose of approximately 1.66 ng of NDs and 0.34 ng of *Neb*-colloostatin) or its application on the cuticle of *T. molitor* (at a dose of approximately 4.15 µg of NDs and 0.85 µg of *Neb*-colloostatin) caused a significant decrease in the phagocytic ability of hemocytes in all studied developmental stages (Fig. 5A). When ND-*Neb*-colloostatin was injected, the percentage of hemocytes phagocytizing latex beads was 86% lower in larvae, 92% lower in pupae and 86% lower in adults than in the controls. When ND-*Neb*-colloostatin was applied topically, the percentage of phagocytes was 84% lower in larvae, 89% lower in pupae and 81% lower in adults than in the controls.

As shown in Fig. 5B, ND injection and topical application (at a dose of 2 ng and 5 µg of NDs per insect, respectively) did not alter the number of nodules formed in the hemocoel of experimentally infected beetles at all stages of development in comparison to the control bacterially challenged larvae, pupae and adults. However, in comparison to the controls, ND-*Neb*-colloostatin decreased nodule formation by 35% in larvae, 48% in pupae and 33% in adults when it was injected into the mealworms at a dose of approximately 1.66 ng of NDs and 0.34 ng of *Neb*-colloostatin. When ND-*Neb*-colloostatin was topically applied to the insects at a dose of approximately 4.15 µg of NDs and 0.85 µg of *Neb*-colloostatin, nodule formation decreased by 32% in larvae, 46% in pupae and 29% in adults (Fig. 5B).

### Phenoloxidase activity assay

**Figure 6:**
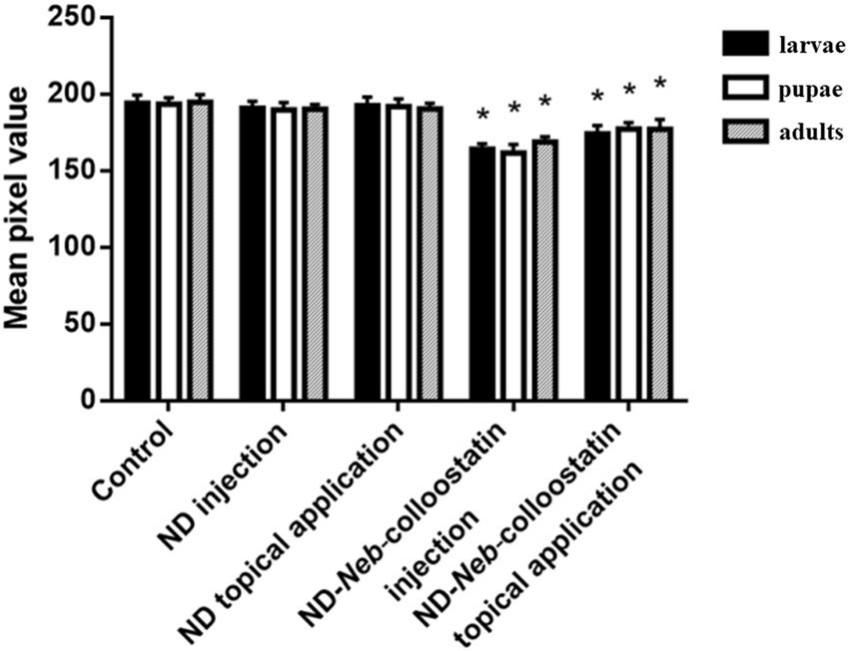
Phenoloxidase activity in the bacterially infected *T. molitor* larvae, pupae and adults following the injection or topical application of NDs or ND-*Neb*-colloostatin. Values shown are means ± S.D.s. Results that significantly differed from the control group at *p* < 0.05 are indicated (*).

We compared the PO activity of ND- and ND-*Neb*-colloostatin-treated and subsequently bacterially challenged beetles at all stages of development in comparison to the saline-treated and bacterially challenged controls (Fig. 6). We observed no significant changes in PO activity in the hemolymph of larvae, pupae and adults irrespective of the application method of NDs. However, the injection of ND-*Neb*-colloostatin solution (at a dose of approximately 1.66 ng of NDs and 0.34 ng of *Neb*-colloostatin) or its application on the cuticle of *T. molitor* (at a dose of approximately 4.15 µg of NDs and 0.85 µg of *Neb*-colloostatin) caused a significant decrease in PO activity in all studied developmental stages. When injected, ND-*Neb*-colloostatin reduced PO activity by 15% in the hemolymph of the larvae, 16% in the hemolymph of the pupae and 13% in the hemolymph of the adults. When topically applied, it reduced PO activity by 10% in the hemolymph of the larvae, 8% in the hemolymph of the pupae and 9% in the hemolymph of the adults.

### Biodistribution of NDs

**Figure 7:**
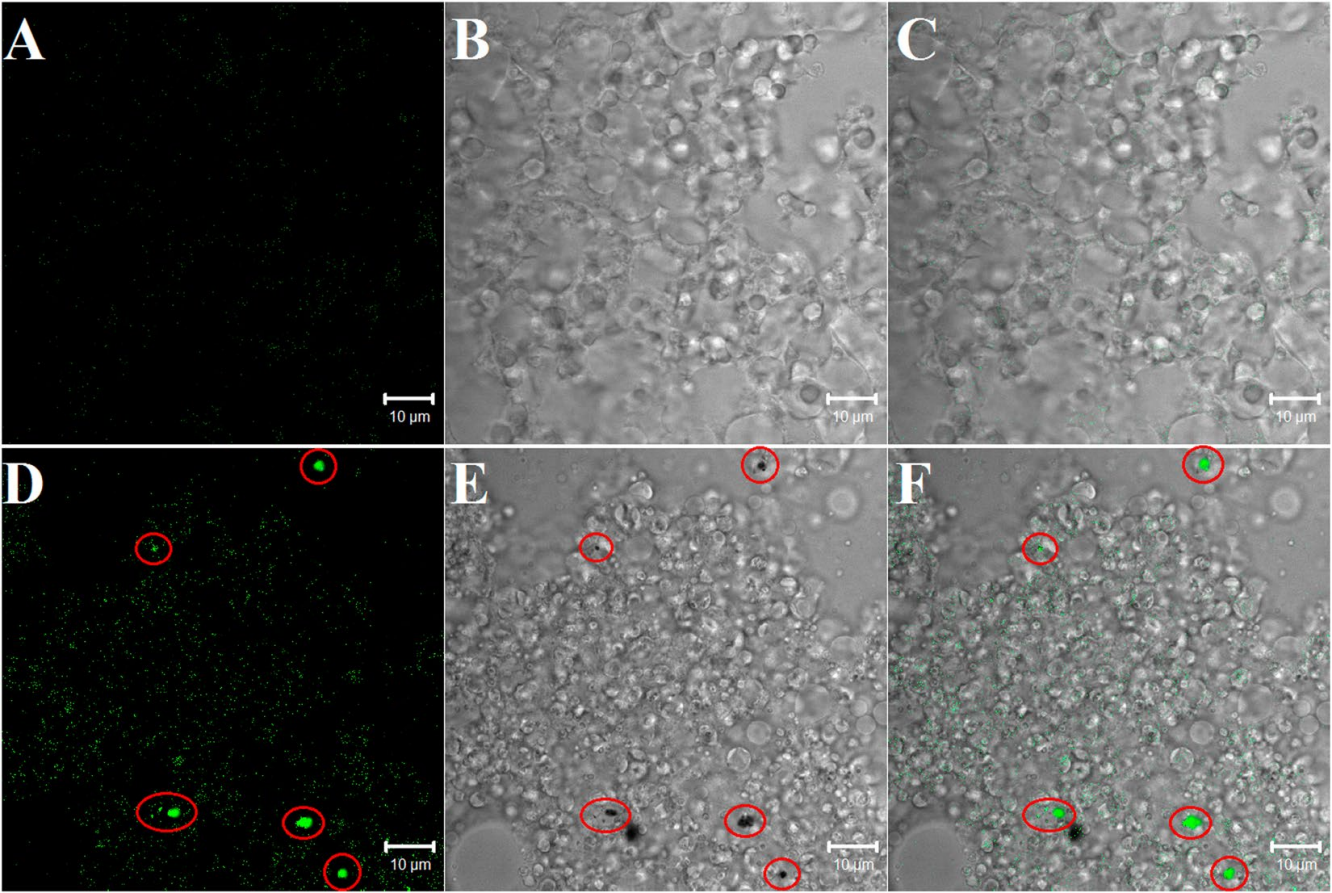
Biodistribution of NDs in the fat body following the exposure of *T. molitor* to FITC-NDs. (**A–C**) Control fat body; (**D–G**) NDs are visible in the fat body cells of insects exposed to 5 µg of FITC-ND for 1 day. The red circles show the colocalization of FITC signals and black dots. Black dots indicate, in the transmitted light, FITC-ND conglomerates.

**Figure 8:**
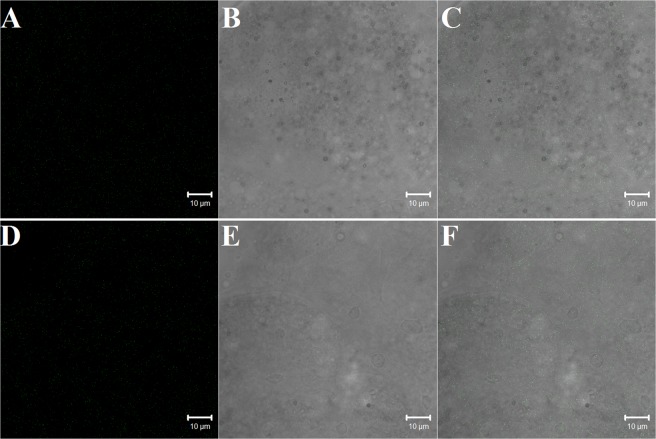
Biodistribution of NDs in Malpighian tubule cells following exposure of *T. molitor* to FITC-NDs. (**A–C**) Control cells of the Malpighian tubules. (**D–F**) There was no colocalization of FITC signals and black dots indicating ND accumulation in the Malpighian tubule cells of insects exposed to 5 µg of FITC-ND for 1 day.

For the *in vivo* testing of ND uptake by cells, mealworms were exposed to FITC-NDs applied on the surface of the mealworm cuticle. We identified large conglomerates of fluorescent NDs in circulating hemocytes (Fig. 2B,C,E,F) and very few conglomerates of NDs in cells of the fat body (Fig. 7D–G). However, we did not detect aggregates of FITC-NDs in the cells of the excretory epithelium of Malpighian tubules (Fig. 8D–F).

## Discussion

This study shows for the first time that NDs easily pass through the insect cuticle, do not interfere with immune responses at the dosages administered, and can be used in insects as a carrier of active insect peptides to reduce the immunity and reproduction of pests.

In most *in vivo* studies of ND cytotoxicity in animal models, this nanoparticle has been administered orally or by injection^5,38,39^. The *in vitro* permeability of NDs through the skin has been studied, and these nanoparticles are recommended as an excellent topical drug delivery system, especially for oxidation- and light-sensitive drugs, due to their improved permeability, higher stability, and minimal safety issues^40^. However, the penetration ability of NDs and the bioactive complexes they carry through the insect’ cuticle and their subsequent *in vivo* effects on the insect have not yet been investigated.

First, we used a very sensitive hemocyte biotest that we previously developed, through which it is possible to detect changes in the morphology, adhesion and viability of hemocytes that can be induced by various biotic and abiotic factors^19,20,29–32^. Using this biotest, we demonstrated that FITC-NDs administered on the cuticle surface passed through this barrier into the hemolymph, where they were immediately phagocytosed by hemocytes. We detected FITC-NDs engulfed in large quantities by hemocytes as visible aggregates under a confocal microscope. This biotest showed that the requirement for ND penetration through the insect cuticle is that the size of the nanoparticles is smaller than the diameter of nanopores, allowing them to pass through the nanopore.

Second, we showed that the NDs applied topically in the doses tested to the surface of the insect cuticle did not induce any cytotoxic changes in hemocytes, as we did not observe any changes in the morphology and adhesion of hemocytes and did not detect any signs of apoptosis in these cells. The phenotype of ND-induced hemocytes was similar to that observed in the control cells. In addition, the results obtained through examination of the number of hemocytes circulating in the hemolymph and *in vivo* immunological bioassays allowing the assessment of hemocyte functionality indicated that at the doses tested, the NDs are not toxic to hemocytes. Upon injection or topical application, NDs did not affect the number of hemocytes circulating in the hemolymph or the ability of hemocytes to phagocytose latex beads or create nodules and did not activate PO in the hemolymph of bacterially infected insects at all developmental stages of *T. molitor*. Our research contradicts a previous study on the cytotoxicity of NDs conducted in another insect model, the house cricket, *Acheta domesticus*^39^. This short-term study in *A. domesticus* showed that the exposure of insects to NDs administered through food (at a dose of 20 µg and 200 µg g^−1^ of food) caused DNA damage in hemocytes and gut cells. In hemocytes, the DNA damage level was dose dependent and was comparable with that induced by the strong prooxidant paraquat^39^. NDs at the tested doses did not change oxidative stress parameters, catalase activity, total antioxidant capacity, or heat shock protein (HSP70) levels in the hemolymph of *A. domesticus*. The discrepancies in the toxicity of NDs in the hemocytes of *T. molitor* and *A. domesticus* may result from the exposure of these insects to nonequivalent doses of NDs, which were lower in the case of the beetle and higher in the case of the cricket. Therefore, we suggest that the NDs are biocompatible with *T. molitor* hemocytes in the doses used in this study and could be employed as transcuticular transporters of the biologically active peptide *Neb*-colloostatin in further research following this study.

The ND biodistribution analysis showed that after the injection and topical exposure of insects to NDs, NDs predominantly accumulated in hemocytes; the fat body was also a target organ for NDs, but ND incorporation into fat body cells was less pronounced than in the case of hemocytes. However, contrary to the results in hemocytes and fat body, we did not detect colocalization of the fluorescent signal (originating from FITC) and the black signal (originating from NDs) in cells of the Malpighian tubules, although we expected such colocalization. This result suggests that the tubule cells do not accumulate large amounts of NDs, but they may successively excrete individual nanoparticles to the interior of tubules; therefore, the confocal signals derived from FITC-NDs are too weak to be detected. We observed green fluorescent signals in cells of the Malpighian tubules under a confocal microscope, but they never colocalized with the black signals originating from NDs and were a result of the autofluorescence of the tracheae closely associated with tubule cells. On the other hand, it is highly likely that the main, and extremely efficient, mechanism of nanoparticle removal is phagocytosis and that hemocytes are the first “scavengers” of NDs that reach the hemocoel of insects. This hypothesis is supported by a small number of signals originating from FITC-NDs detected in the mealworm fat body. The question arises, however, of what will happen to the phagocytes and the ND charge inside them; will these circulating phagocytes settle, adhere to tissues and organs of insect, and create nodule-like structures or, in the long term, will they undergo apoptosis, and will the resulting apoptotic bodies with nanoparticle deposits will be phagocytosed by subsequent hemocytes? The explanation of this issue requires further detailed research.

Our previous work has shown that *Neb*-colloostatin or its more potent analogs injected into *T. molitor* in pico- and nanomolar doses exert strong pro-apoptotic activity in hemocytes in *T. molitor* adults^19,20^; hence, caspase activation induced by these peptides can serve as a biochemical marker of their biological activity in insect hemocytes. Proving that NDs pass through the cuticle was crucial for the implementation of further tests to demonstrate that these nanoparticles can be used as a transcuticular peptide carrier. We functionalized ND with *Neb-*colloostatin, and showed that the administration of ND-*Neb-*colloostatin to the cuticle surface resulted in significant changes in morphology and viability and a marked increase in apoptotic activity in *T. molitor* hemocytes, while neither ND or *Neb-*colloostatin applied topically on the cuticle altered the viability of these cells. This finding clearly indicates that we succeeded in introducing *Neb*-colloostatin complexed with NDs from the insect body surface to the hemolymph and in inducing apoptosis in the hemocytes, which is the specific effect of *Neb*-colloostatin in insects. In turn, the lack of hemocytotoxic effects of *Neb*-colloostatin after its administration on the cuticle indicates that the peptide cannot itself pass through the hydrophobic barrier of the cuticle and penetrate the cavity of the insect body.

Recently, it has been demonstrated that the NDs and ND-poly-D-lysine can efficiently deliver a functional protein in HeLa cells and in *Drosophila*^41^. It has been shown that oral administration of ND-poly-D-lysine-RNase induced apoptosis in enterocytes and increased the number of stem cells in the gut of *Drosophila*, whereas NDs coated with poly-D-lysine and conjugated to BSA did not cause apoptosis in intestinal cells, suggesting the biocompatibility of these nanoparticles. Moreover, the positively charged ND-poly-D-lysine-protein complex was shown to discard the fusion with the early endosome, thereby avoiding protein degradation in the lysosome. This result may suggest, that in hemocytes exposed to the ND-*Neb*-colloostatin, there may be a similar mechanism to avoid of the peptide degradation.

We also showed that the hemocytotoxic activity of *Neb*-colloostatin introduced to the hemocoel in complex with NDs *via* injection or through the cuticle has serious implications for *T. molitor* immune defense. In insects, hemocytes play a key role in the immune response, including cellular and humoral defense. The cellular immune response involves the functions of hemocytes in the phagocytosis of abiotic and biotic targets, encapsulation of large pathogens and nodule formation to entrap a large number of small pathogens^42^. Nodulation is generally considered the most important defense mechanism against pathogen infections in insects and other invertebrates. It is a complex process that is initiated by the activation of a substantial number of hemocytes circulating in the hemolymph, which change from non-adhesive to adherent cells with a tendency to form aggregates surrounding bacteria, fungi and viruses^32,43^.

Induction of release of properoxidase from hemocytes to the hemolymph by invaders and then activation of a serine protease cascade leads to the production of active phenoloxidase. Phenoloxidase is an enzyme responsible for localized hemolymph coagulation and melanin deposition around damaged tissues or the isolation of microorganisms inside nodules produced by hemocytes^42,44^. Therefore, maintaining the correct number of healthy hemocytes is crucial for the effective functioning of the insect’s immune system and, consequently, for the insect’s survival^19,42^. We showed that the result of the hemocytotoxic action of *Neb*-colloostatin introduced in complex with NDs to the body of the insects was a significant reduction in the number of hemocytes circulating in the hemolymph of larvae, pupae and adults of *T. molitor*. A short-term consequence of the pronounced peptide-induced drop in the number of hemocytes was a significant decrease in latex bead phagocytosis, whereas a long-term effect of hemocyte depletion was a substantial decrease in nodule formation and phenoloxidase activity in all stages of *T. molitor* development. In the present work, we showed that *Neb*-colloostatin is a very active peptide that can disturb the innate resistance of insects. In our previous study, we detected nine additional hemocytotoxic analogs of *Neb*-colloostatin^20^, and it can be assumed that these analogs will show stronger immunoinhibitory and gonadoinhibitory effects in insects than the native peptide.

## Conclusion

The present study is the first to indicate that NDs can transfer the peptide *Neb*-colloostatin through the cuticle to the hemolymph of insects, where it exerts a proapoptotic effect on hemocytes. The peptide introduced *via* this route decreases the number of hemocytes circulating in the hemolymph and impairs the innate immune response of the insect. NDs themselves do not exert cytotoxic effects in hemocytes and do not disturb their response to the introduction of latex beads into the body of the insect or bacterial infection. Due to the high phagocytic activity of the hemocytes, the NDs are efficiently removed from the hemolymph, which probably protects the cells of other tissues and organs from potential unfavorable effects of these nanoparticles. It is possible that research on the transcuticular transport of active insect peptides that can interfere with the immune response and reproduction of insects will contribute to the development of new bioinsecticides that will be safe for other organisms and the environment.

## Data Availability

The datasets used during the current study are available from the corresponding author upon reasonable request.

## References

[1] Mochalin VN, Shenderova O, Ho D, Gogotsi Y (2012). The properties and applications of nanodiamonds. Nat Nanotechnol.

[2] Vaijayanthimala V (2012). The long-term stability and biocompatibility of fluorescent nanodiamond as an *in vivo* contrast agent. Biomaterials.

[3] Vaijayanthimala V (2015). Nanodiamond-mediated drug delivery and imaging: challenges and opportunities. Exp Opin Drug Deliv.

[4] Nappi MR, Christensen BM (2005). Melanogenesis and associated cytotoxic reactions, application to innate immunity. Insect Biochem Mol Biol.

[5] Karpeta-Kaczmarek J (2018). Chronic toxicity of nanodiamonds can disturb development and reproduction of *Acheta domesticus* L. Environ Res.

[6] Kurantowicz N (2017). Toxicity studies of six types of carbon nanoparticles in a chicken-embryo model. Int J Nanomedicine.

[7] Tasat DR (2016). Biokinetics and tissue response to ultrananocrystalline diamond nanoparticles employed as coating for biomedical devices. J Biomed Mater Res Appl Biomater.

[8] Yuan Y (2010). Pulmonary toxicity and translocation of nanodiamonds in mice. Diam Relat Mater.

[9] Mytych J (2015). Nanodiamond-induced increase in ROS and RNS levels activates NF-κB and augments thiol pools in human hepatocytes. Diam Relat Mater.

[10] Huang Y-A (2014). The effect of fluorescent nanodiamonds on neuronal survival and morphogenesis. Scientific Reports.

[11] Marcon L, Riquet F, Szunerits S (2010). Cellular and *in vivo* toxicity of functionalized nanodiamond in *Xenopus* embryos. J Mater Chem.

[12] Mendonça E (2011). Effects of diamond nanoparticle exposure on the internal structure and reproduction of *Daphnia magna*. J Hazard Mater.

[13] Rojas S (2011). Biodistribution of amino-functionalized diamond nanoparticles. *In vivo* studies based on 18F radionuclide emission. ACS Nano.

[14] Cid A (2015). Oxidative stress and histological changes following exposure to diamond nanoparticles in the freshwater Asian clam *Corbicula fluminea* (Müller, 1774). J Hazard Mater.

[15] Lin Y-C (2016). Nanodiamond for biolabelling and toxicity evaluation in the zebrafish embryo *in vivo*. J Biophoton.

[16] Bylemans D (1995). *Neb*-colloostatin, a second folliculostatin of the grey fleshfly *Neobellieria bullata*. Eur J Bioch.

[17] Wasielewski O, Rosiński G (2007). Gonadoinhibitory effects of *Neb*-colloostatin and *Neb*-TMOF on ovarian development in the mealworm, *Tenebrio molitor* L. Arch Insect Biochem Physiol.

[18] Czarniewska E (2014). The natural insect peptide *Neb*-colloostatin induces ovarian atresia and apoptosis in the mealworm *Tenebrio molitor*. BMC Dev Biol.

[19] Czarniewska E (2012). The pro-apoptotic action of the peptide hormone *Neb*-colloostatin on insect haemocytes. J Exp Biol.

[20] Kuczer M (2013). The pro-apoptotic action of new analogs of the insect gonadoinhibiting peptide *Neb*-colloostatin: Synthesis and structure activity studies. Peptides.

[21] Abernathy RL (1996). Induction of moth sex pheromone production by topical application of an amphiphilic pseudopeptide mimic of pheromonotropic neuroepeptides. Proc Natl Acad Sci USA.

[22] Nachman RJ (1996). Potent pheromonotropic/myotropic activity of a carboranyl pseudotetrapeptide analog of the insect pyrokinin/PBAN neuropeptide family administered via injection and topical application. Peptides.

[23] Teal PEA, Nachman RJ (1997). Prolonged pheromonotropic activity of pseudopeptide mimics of insect pyrokinin neuropeptides after topical application or injection into a moth. Reg Peptides.

[24] Teal PEA, Meredith JA, Nachman RJ (1999). Comparison of rates of penetration through insect cuticle of amphiphilic analogs of insect pyrokinin neuropeptides. Peptides.

[25] Nachman RJ, Teal PEA, Ujvary I (2001). Comparative topical phermonotropic activity of insect pyrokinin/PBAN amphiphilic analogs incorporating different fatty and/or cholic acid components. Peptides.

[26] Moret Y, Moreau J (2012). The immune role of the arthropod exoskeleton. ISJ.

[27] Wigglesworth VB (1948). The structure and deposition of the cuticle in the adult mealworm *Tenebrio molitor* L. (Coleoptera). Q J Micros Sci.

[28] Yusko EC (2011). Controlling the translocation of proteins through nanopores with bioinspired fluid walls. Nat Nanotechnol.

[29] Kuczer M (2016). Novel analogs of alloferon: synthesis, conformational studies, pro-apoptotic and antiviral activity. Bioorg Chem.

[30] Kadej A (2016). High stability and biological activity of the copper(II) complexes of alloferon 1 analogues containing tryptophan. J Inorg Biochem.

[31] Kowalik-Jankowska T (2017). Copper(II) complexes of the Neb-colloostatin analogues containing histidine residue structure stability biological activity. Polyhedron.

[32] Czarniewska E (2018). The long-term immunological effects of alloferon and its analogues in the mealworm *Tenebrio molitor*. Insect Sci.

[33] Plata-Rueda A (2017). Insecticidal activity of garlic essential oil and their constituents agents against the mealworm beetle, *Tenebrio molitor* Linnaeus (Coleoptera: Tenebrionidae). Sci Rep.

[34] Fields GB, Noble RL (1990). Solid phase peptide synthesis utilizing 9-fluorenylmethoxycarbonyl amino acids. Int J Pept Protein Res.

[35] Kuczer M, Rosiński G, Konopińska D (2007). Insect gonadotropic peptide hormones: some recent developments. J Pept Sci.

[36] Sorrentino, R., Small, C. N. & Govid S. Quantitative analysis of phenol oxidase activity in insect hemolymph. Biotechniques **32**, 815–6, 818, 820, 822–3 (2002). 10.2144/02324st08 11962604

[37] Trauer U, Hilker M (2013). Parental legacy in insects: variation of transgenerational immune priming during offspring development. PlosOne.

[38] Mohan N (2010). *In vivo* imaging and toxicity assessment of fluorescent nanodiamonds in *Caenorhabditis elegans*. Nano Lett.

[39] Karpeta-Kaczmarek J (2016). Effects of short-term exposure of *Acheta domesticus* to nanodiamonds in food: DNA damage but no histological alternation in tissues. Carbon.

[40] Lim DG (2016). Comprehensive evaluation of carboxylated nanodiamonds as topical drug delivery system. Int J Nanomedicine.

[41] Hu X (2017). Nanodiamonds mediate oral delivery of proteins for stem cell activation and intestinal remodeling in *Drosophila*. ACS Appl Mater Interfaces.

[42] Lavine MD, Strandt MR (2002). Insect hemocytes and their role in immunity. Insect Biochem Mol Biol.

[43] Satyavathi VV, Minz A, Nagaraju J (2014). Nodulation: An unexplored cellular defense mechanism in insects. Cell Signal.

[44] Nappi AJ, Christensen BM (2005). Melanogenesis and associated cytotoxic reactions: applications to insect innate immunity. Insect Biochem Mol Biol.

